# Adhesion of renal carcinoma cells to endothelial cells depends on PKCμ

**DOI:** 10.1186/1471-2407-10-183

**Published:** 2010-05-06

**Authors:** Walburgis Brenner, Silke Beitz, Elke Schneider, Frank Benzing, Ronald E Unger, Frederik C Roos, Joachim W Thüroff, Christian Hampel

**Affiliations:** 1 Department of Urology, University Medical Center Mainz, Mainz, Germany; 2 Institute of Pathology, University Medical Center Mainz, Mainz, Germany

## Abstract

**Background:**

The formation of metastases includes the separation of tumor cells from the primary tumor, cell migration into subendothelial tissue and cell proliferation in secondary organ. In this process, cell adhesion of tumor cells to the endothelium is an essential requirement for formation of metastases. Protein kinase C (PKC) regulates adhesion and proliferation. To identify a relation between PKC isoforms and tumor progression in renal cell carcinoma (RCC), the influence of PKC isoforms on cell adhesion and proliferation, and possible influences of integrins were analyzed in RCC cells.

**Methods:**

The experiments were performed in the RCC cell lines CCF-RC1 and CCF-RC2 after pre-incubation (16 h) with the PKC inhibitors GF109203X (inhibits PKCα, βI, βII, γ, δ and ε), GÖ6976 (inhibits PKCα, βI and μ), RO31-8220 (inhibits PKCα, βI, βII, γ and ε) and rottlerin (inhibits PKCδ). Cell adhesion was assessed through adherence of RCC cells to an endothelial monolayer. Cell proliferation was analyzed by a BrdU incorporation assay. The expression of β1 integrins was analyzed by flow cytometry.

**Results:**

In CCF-RC1 cells, cell adhesion was significantly reduced by GÖ6976 to 55% and by RO31-8220 to 45% of control. In CCF-RC2 cells, only GÖ6976 induced a significant reduction of cell adhesion to 50% of control levels. Proliferation of both cell lines was reduced by rottlerin to 39% and 45% of control, respectively. The β1 integrin expression on the cell surface of CCF-RC1 and CCR-RC2 cells was decreased by RO31-8220 to 8% and 7% of control, respectively. β2 and β3 integrins were undetectable in both cell lines.

**Conclusions:**

The combination of the PKC inhibitors leads to the assumption that PKCμ influences cell adhesion in CCF-RC1 and CCF-RC2 cells, whereas in CCF-RC1 cells PKCε also seems to be involved in this process. The expression of β1 integrins appears to be regulated in particular by PKCε. Cell proliferation was inhibited by rottlerin, so that PKCδ might be involved in cell proliferation in these cells.

## Background

Formation of metastases includes the separation of single cells from the primary tumor, migration into the extracellular matrix, blood vessel invasion, adhesion to endothelium, migration through the endothelium and growth in a secondary organ [1]. During extravasation into the secondary organ, tumor cells seem to undergo the same mechanisms as leukocytes in inflammatory processes. After a loose contact to endothelial cells, integrins on the cell surface of leukocytes become activated by a chemokine induced inside-out signaling sought by endothelial cells [2] or by direct cell-cell contact [3]. Activated integrins, in particular β1, β2 and β3 integrins, mediate a firm adhesion to endothelial cells by binding their ligands such as ICAM, VCAM, PECAM or other integrins [4-6] leading to transendothelial migration. In the process of metastases, the adhesion of tumor cells to endothelial cells has also been shown to be mediated by integrins. The tumor cells bind their ligands, located on the cell surface of endothelial cells, leading to a firm adhesion, and subsequently to transendothelial migration. *In vitro *experiments showed a major importance in the binding of α4β1 integrin to VCAM in several tumor entities in tumor cell adhesion [7,8]. Furthermore, α6β1, αvβ1 and αvβ3 integrins have been shown to be involved in tumor cell-endothelial cell adhesion [9-11].

In renal cell carcinoma, an important role has also been demonstrated for β1 integrins [12,13]. The function of integrins can rapidly be changed by altering their binding affinity for ligands through inside-out signaling. Inside-out signaling induces a conformational change from the cytoplasmic domains in the direction of the extracellular binding site, in response to intracellular signaling events. Signaling molecules involved in inside-out signaling of integrins are G proteins, Ca^2+^, phospholipase, tyrosine kinase, CaM kinase II, and protein kinases C (PKCs) [14-16]. The activation pathway on integrins by PKC includes RACK (receptor for activated C kinase), which binds to the β subunit of integrins [17]. PKC modulation results in an alteration of the integrin avidity and affinity [18]. In addition to the activity of integrins, PKC regulates the integrin expression on the cell surface [19,20]. These reports demonstrate the interaction between PKC and integrins.

The family of PKC comprises phospholipid dependent serine/threonine protein kinases deriving from different PKC genes, and from alternative splicing of a single transcript [21]. Up to 10 distinct family members have been discovered in mammalian cells, which are classified into Ca^2+^-dependent conventional cPKC isoforms α, βI, βII and γ, Ca^2+^-independent novel nPKCs δ, ε, η and θ, and the atypical aPKCs λ/τ and ζ. PKCμ/PKD, a Ca^2+ ^independent PKC with a unique substrate specificity which differs from the PKC isoforms [22], has primary been related to the PKC family, but cannot be attributed as a member of the PKC family. In contrast to the PKC family, which belongs to the AGC group (PKA, PKG, PKC), PKCμ belongs to the CAMK group (Calcium/calmodulin-dependent protein kinase) [23,24]. The expression patterns of PKC isoforms differ between tissues and the subcellular distribution of the isoforms varies depending on cell type and physiological condition [25-27], so that an overexpression of the same PKC isoform in different cells may result in opposite biological effects, depending on the cell type [28,29].

To date, little is known about the isoform specific role of PKC in metastasis of renal cancer. To answer this question, we investigated the influence of different-isoform specific PKC inhibitors on the metastatic behavior of renal carcinoma cells *in vitro*. Consequently, the involvement of PKC in the adhesion of renal tumor cells, as well as cell proliferation and the expression of integrins was analyzed.

## Methods

### Cells and cell culture

The human renal carcinoma cell lines CCF-RC1 and CCF-RC2 were used for all experiments. CCF-RC1 was isolated from a high grade pleomorphic renal cell carcinoma (nuclear grade 3) [30,12], CCF-RC2 was cultured from the effluent of the same kidney. Cell culture was maintained in RPMI 1640 with L-glutamine (Gibco, Eggenstein, Germany), supplemented with 10% fetal calf serum and 0.5% penicillin/streptomycin (Gibco, Eggenstein, Germany). The cells were incubated at 37°C in a humidified atmosphere containing 5% CO_2 _in air. Cells in the present study were used between passage 20 and 30.

For cell adhesion experiments, human umbilical vein endothelial cells (HUVEC) were isolated as previously described [31,32]. Briefly, intact segments of 1 to 3-days-old umbilical cords were drained, and the remaining blood was rinsed from the umbilical vein with phosphate buffered saline (PBS) via a blunt cannula attached at one end of the vessel. The open end of the cord was sealed and the cord distended with 1 mg/ml collagenase II and incubated at room temperature for 30 min. The cord was massaged and cut, and the collagenase digest collected. The cells were washed, seeded into fibronectin-coated flasks (1 μg human fibronectin per cm^2 ^flask) and grown in Medium 199 media supplemented with 20% fetal calf serum (FCS), 25 μg/ml heparin and 25 μg/ml endothelial cell growth supplement (ECGS). Cells were subcultured by trypsin-EDTA treatment and used until the fifth passage in the experiments.

### Treatment of renal tumor cells with PKC inhibitors

The renal tumor cells CCF-RC1 and CCR-RC2 were incubated with the PKC inhibitors GF109203X (inhibits PKCα, βI, βII, γ, δ and ε), GÖ6976 (inhibits PKCα, βI and μ), RO31-8220 (inhibits PKCα, βI, βII, γ and ε) (concentrations of 1, 2 and 5 μM) and rottlerin (inhibits PKCδ) (concentrations of 1, 5 and 10 μM, all from Calbiochem, La Jolla, USA) in serum free medium for 16 hours at 37°C in a humidified atmosphere containing 5% CO_2 _in air. The IC_50 _values of the inhibitors against PKC isoforms are listed in Table 1. Serum free culture medium without inhibitors served as a control. To analyze toxic effects of PCK inhibitors, renal tumor cells were incubated with PKC inhibitors as described above, each of them fourfold.

**Table 1:** IC_50 _values for GF109203X, GÖ6976, RO31-8220 (each nM) and Rottlerin (μM) for inhibition of PKC isoforms (from: Goekjian and Jirousek, 1999 [35] and Gschwendt et al., 1994 [62])

Inhibitors	PKC isoforms								
	**α**	**βI**	**βII**	**γ**	**δ**	**ε**	**η**	**ξ**	**μ**

GF109203X [nM]	14	18	16	20	210	132		5800	2000
GÖ6976 [nM]	2.3	6.2			>10000	>10000	685	>10000	20
RO31-8220 [nM]	5	24	14	27		24			
Rottlerin [μM]	30	42		40	3-6	100	82	100	

### MTT cell vitality assay

The mitochondrial activity of the cells was assessed by a MTT assay kit (Sigma, Deisenhofen, Germany). In this assay the vitality of living cells was measured via mitochondrial dehydrogenases. The yellow MTT (3-[4,5-dimethylthiazol-2-yl]2,5-diphenyl tetrazolium bromide) was reduced to a blue formazan product by mitochondrial activity. Briefly, after incubation of the cells with PKC inhibitors, culture medium was replaced by MTT solution (0.5 mg/ml in phenol red free medium) and incubated for two hours at 37°C. Afterwards, formazan crystals were dissolved by MTT solubilization solution and measured spectrophotometrically at a wavelength of 570 nm (reference wavelength 690 nm).

### Cell adhesion assay

To analyze the adhesion of renal tumor cells to an endothelial monolayer, HUVEC (1.5 × 10^4 ^cells/well) were seeded into a 96 well plate coated with 1.5 mg/cm^2 ^gelatine. Cells were allowed to grow to a confluent monolayer within three days. PKC inhibitor-treated and control CCF-RC1 cells were labeled by 10 μM BrdU (Bromodeoxyuridine) for 30 min at 37°C. A cell suspension containing 10^4 ^cells was added to the endothelial cell layer under presence of the particular PKC inhibitor and incubated at 37°C in a humidified atmosphere containing 5% CO_2 _in air. After two hours, non-adherent cells were removed by washing with PBS. To normalize the level of BrdU per cell, a defined number of 10^4 ^BrdU labeled tumor cells were centrifuged in parallel into a 96 well plate. The relative amount of BrdU per well was quantified as described in the next section. Each experiment was performed eightfold and repeated three times.

### Analysis of cell proliferation

To study the effect of PKC inhibitors on proliferation, a colorimetric BrdU incorporation assay (Roche, Mannheim, Germany) was performed. Each 3 × 10^3 ^cells were seeded in triplicate into a 96 well plate and treated in triplicate by PKC inhibitors in serum free medium. BrdU solution (10 μM) was added to the cells and incubated for two hours at 37°C in a humidified atmosphere containing 5% CO_2 _in air. After removing the culture medium, the cells were fixed and the DNA was denatured in one step by adding fixDenat solution. Incorporated BrdU was detected by an anti-BrdU-POD antibody. The immune complex was detected by a subsequent substrate reaction and quantified by measuring the absorbance at 450 nm (reference wavelength 690 nm).

### Western blot analyses

For preparation of protein extracts, semiconfluent CCF-RC1 and CCF-RC2 members were rinsed twice with ice-cold phosphate-buffered saline (PBS) and scraped off the dish with a rubber policeman in lysis buffer on ice (20 mM HEPES, pH 7.7, 0.2 M NaCl, 1.5 mM MgCl2, 0.4 mM EDTA, 1% Triton X-100, 0.5 mM DTT, 100 μg/ml leupeptin, 100 μg/ml aprotinin, 10 mM benzamidine, 2 mM phenylmethylsulphonyl fluoride, 20 mM β-glycerophosphate and 0.1 mM sodium-orthovanadate) [33]. Cell lysate was centrifuged at 14,000 rpm at 4°C for 20 min. Protein concentrations of the supernatants were determined using Bicinchoninic acid reagents. Thirty μg protein/lane protein extract of the cells was separated by SDS-PAGE (sodium dodecyl sulfate-polyacrylamide gel electrophoresis) in 10% acrylamide gels and transferred by semi-dry blotting onto polyvinylidene fluoride membranes (PVDF, Immobilon P, Millipore, Bedford, MA). The membrane was incubated with blocking solution (Roth, Karlsruhe, Germany) for 1 h before incubation with the primary antibodies against the PKCδ (78 kDa), PKCε (83 kDa), PKCμ/PKD1 (115 kDa), PDK2 (105 kDa) and PKCν/PDK3 (100 kDa) (all 1:300, Santa Cruz, Heidelberg, Germany) overnight at 4°C. After washing three times, the membrane was incubated with horseradish peroxidase-conjugated goat anti-rabbit secondary antibodies (Dako, Hamburg, Germany) for 1 h at room temperature. The bound antibodies were visualized by enhanced chemiluminescence (ECL, NEN Lifescience, Boston, USA) detection system using Fuji medical X-ray film.

### Flow cytometry

The integrin surface expression of untreated as well as PKC inhibitor treated tumor cells was quantified by flow cytometry. Briefly, cells were harvested in a 0.02% EDTA solution and incubated with a monoclonal FITC labeled anti-CD29 (β1 chain of integrins), anti-CD18 (β2 chain of integrins), anti-CD61 (β3 chain of integrins) or an isotypic control immunoglobuline (all Immunotech, Hamburg, Germany), respectively, for 15 min, at 4°C in darkness. Then, integrin expression was quantified on a flow cytometer (BP Calibur, Becton Dickinson, Heidelberg, Germany).

### Statistical analysis

For statistical confirmation, three or four independent adhesion and proliferation experiments, each four- to eightfold, were performed respectively. A mean value and a standard error were calculated from the results. For significance analyses, SPSS 17.0 software was applied. Differences in cell vitality, proliferation and adhesion were performed using the Mann-Whitney test. Differences were considered statistically significant at p < 0.05.

## Results

### Cell vitality after PKC inhibition

**Figure 1:**
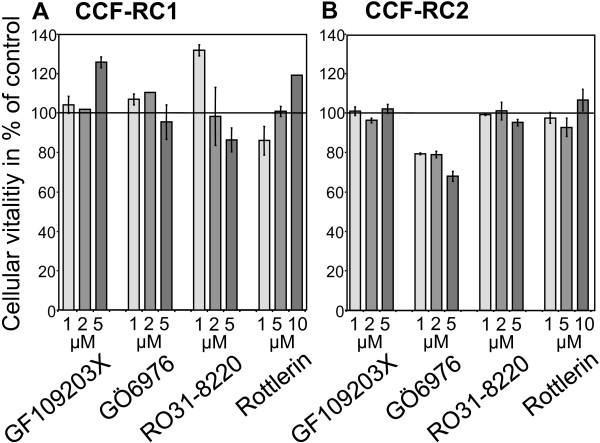
**Cell vitality after PKC inhibition**. CCF-RC1 (A) and CCF-RC2 (B) cells were treated with PKC isoform specific inhibitors GF109203X, GÖ6976, RO31-8220 and rottlerin in different concentrations, cell vitality was determined by MTT assay. Only treatment of CCF-RC2 cells with GÖ6976 resulted in a slight reduction of cell vitality. The columns and bars represent mean value and standard error in % of control (untreated cells).

Renal tumor cell vitality was determined by MTT assays. With exception of GÖ6976 in CCF-RC2 cells, treatment of renal cancer cells with the PKC inhibitors GF109203X, GÖ6976, RO31-8220 (1, 2 and 5 μM) and rottlerin (1, 5 and 10 μM) did not influence cell vitality. Treatment of CCF-RC2 cells with GÖ6976 resulted in a non-significant reduced vitality to 68% of control (Figure 1).

### Cell proliferation after PKC inhibition

**Figure 2:**
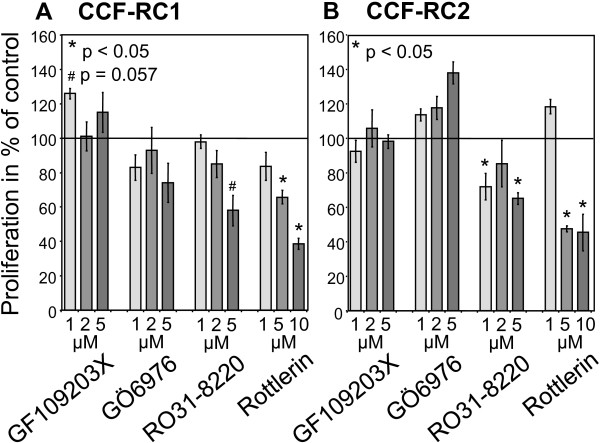
**Cell proliferation after PKC inhibition**. CCF-RC1 (A) and CCF-RC2 (B) cells were treated with the PKC isoform specific inhibitors GF109203X, GÖ6976 and RO31-8220 in concentrations between 1 μM and 5 μM, and with Rottlerin in concentrations between 1 μM and 10 μM. The proliferation value is determined as a percentage of the proliferation of control (untreated cells). The columns and bars represent mean value and standard error in % of control.

To assess the influence of PKC inhibition on cell proliferation, CCF-RC1 and CCF-RC2 cells were incubated with the PKC inhibitors GF109203X, GÖ6976, RO31-8220 and rottlerin in concentrations as described. Proliferation was determined by BrdU incorporation. In CCF-RC1 and CCF-RC2 cells we observed reduced cell proliferation after treatment with RO31-8220 and rottlerin in a concentration dependent manner. RO31-8220 led to 58% (CCF-RC1, p = 0.057) and 68% (CCF-RC2, p = 0.012) of control cell proliferation. After rottlerin treatment, cell proliferation was reduced to 37% p = 0.029) and 45% (p = 0.012) of control measurements in CCF-RC1 and CCF-RC2 cells, respectively. In CCF-RC1 cells, also treatment with GÖ6976 resulted in a slightly reduced cell proliferation to 74% of control (p = 0.200, Figure 2).

### Cell adhesion of PKC inhibitor treated renal tumor cells to endothelial cells

**Figure 3:**
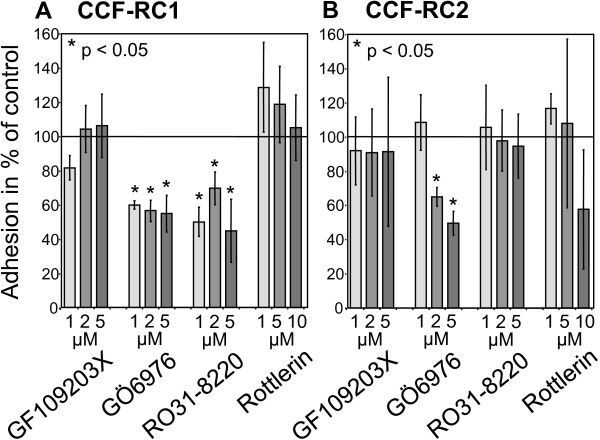
**Cell adhesion to endothelial cells after PKC inhibition**. Adhesion of CCF-RC1 (A) and CCF-RC2 (B) cells to a monolayer of human umbilical endothelial cells (HUVEC) was measured after treatment with the PKC isoform specific inhibitors GF109203X, GÖ6976 and RO31-8220 in concentrations between 1 μM and 5 μM, and with Rottlerin in concentrations between 1 μM and 10 μM. The adhesion value is determined as a percentage of the adhesion of control (untreated cells). The columns and bars represent mean value and standard error in % of control.

For investigation of the adhesion of renal carcinoma cells to endothelial cells, CCF-RC1 and CCF-RC2 cells were treated with PKC inhibitors GF109203X, GÖ6976, RO31-8220 and rottlerin as described and the adhesion of these cells to a monolayer of HUVECs was determined. In CCF-RC1 cells, incubation with GÖ6976 and RO31-8220 resulted in a concentration dependent inhibition of cell adhesion to 55% (p = 0.029) and 45% (p = 0.029) of control cells, respectively. In contrast, CCF-RC2 cells showed a reduced tumor cell - endothelial cell adhesion after treatment also with GÖ6976 (50% of control, p = 0.029), whereas RO31-8220 resulted in no alteration of cell adhesion (Figure 3).

### Influence of PKC inhibitors on the expression of β1 integrins

**Figure 4:**
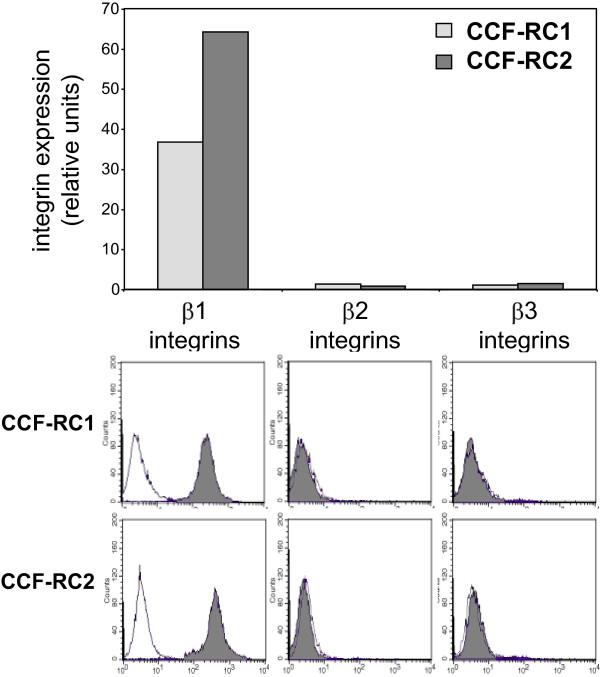
**Expression of β1, β2 and β3 integrins**. Integrin expression was analyzed in untreated CCF-RC1 and CCF-RC2 cells by flow cytometry. The columns represent mean values of 10 000 counted cells. For control, an isotype specific IgG control was used. Only β1 integrins were expressed in both cell lines.

**Figure 5:**
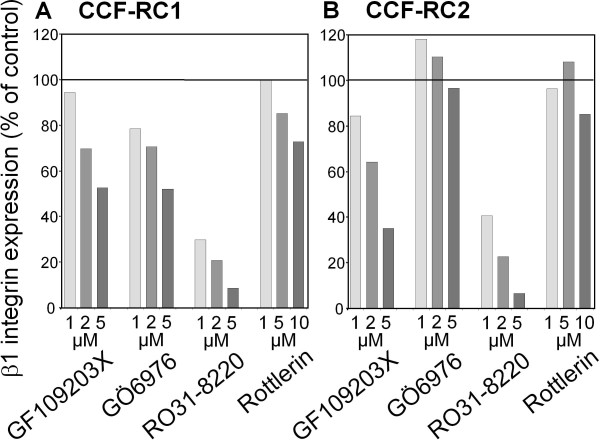
**Expression of β1 integrins after PKC inhibition**. CCF-RC1 (A) and CCF-RC2 (B) cells were treated with the PKC isoform specific inhibitors GF109203X, GÖ6976 and RO31-8220 in concentrations between 1 μM and 5 μM, and with Rottlerin in concentrations between 1 μM and 10 μM, integrin expression was determined by flow cytometry. The columns represent mean value of 10 000 counted cells in % of control (untreated cells).

Since tumor cell adhesion to endothelial cells by integrins is mediated in the first line by β1, β2 and β3 integrins [34], the expression of these integrins was determined by flow cytometry analysis. Both cell lines, CCF-RC1 and CCF-RC2 cells, showed a clear expression of β1 integrins, whereas neither β2 nor β3 integrins were expressed in these renal tumor cells (Figure 4). In CCF-RC1 cells, β1 integrin levels were impaired by all PKC inhibitors used in altered extent. GF109203X induced a reduction of β1 integrin expression to 53%, GÖ6976 to 52%, RO31-8220 to 8% and rottlerin to 73% as compared to controls, respectively. In CCF-RC2 cells, only GF109203X and RO31-8220 treatment resulted in a 35% and 7% reduced β1 integrin expression as compared to control expression levels (Figure 5). In our experiment, RO31-8220 was the strongest inhibitior of β1 integrins.

### Expression of PKCδ, PKC and, PKCμ in CCF-RC2 cells and PKD2 and PKD3 in both cell lines

**Figure 6:**
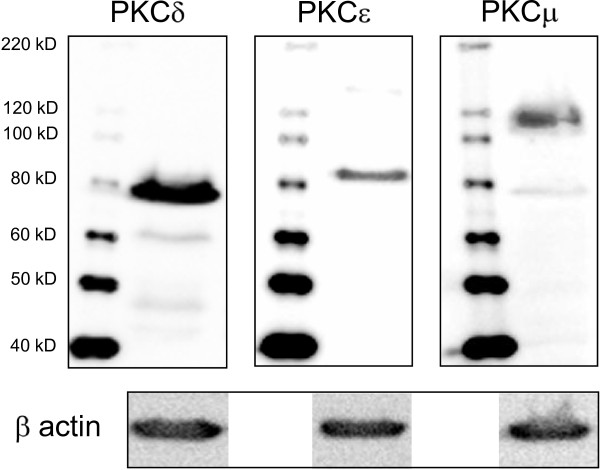
**Expression of PKCδ, ε and μ in CCF-RC2 cells**. Expression of PKCδ (78 kDa), ε (83 kDa) and μ (115 kDa) in CCF-RC2 cell was determined by Western blot (30 μg protein extract per lane). β-actin was used as loading control. All three PKC isoforms are expressed in CCF-RC2 cells.

In former investigations we showed an expression of PKCδ, ε and μ in CCF-RC1 cells [19]. Since these three isoforms seem to be relevant in metastatic processes of renal cancer cells, we analyzed their expression in CCF-RC2 cells. Furthermore, PKC2 and 3 were analyzed in both cell lines. Our Western blot analyses showed a strong expression of PKCδ and, to a lesser extent, PKCε and μ in CCF-RC2 cells (Figure 6). PKC2 and 3 (PKCν) were not detectable in either cell line (data not shown).

## Discussion

Renal cell carcinoma is a tumor entity with a high risk for metastases and consequently carries a poor prognosis. Therefore, investigations are based on the analysis of metastatic mechanisms. Adhesion of tumor cells to the endothelium of blood vessels during the process of extravasation is one of the most important prerequisites for the formation of metastasis. We investigated the influence of various PKC isoforms on the regulation of endothelial cell adhesion of the renal carcinoma cell lines CCF-RC1 and CCF-RC2. The cells were incubated with PKC isoform specific inhibitors GF109203X (inhibits PKCα, βI, βII, γ, δ and ε), GÖ6976 (inhibits PKCα, βI and μ), RO31-8220 (inhibits PKCα, βI, βII, γ and ε) and rottlerin (inhibits PKCδ) [35]. Afterwards we quantified cell adhesion to endothelial cells, cell proliferation and the expression of β1, β2 and β3 integrins. In contrast to our former investigations [36], we pre-incubated the cells with the PKC inhibitors for 16 hours. This long-time incubation not only can lead to regulatory response but also allows intracellular changes in gene expression.

In CCF-RC1 cells treatment with GÖ6976 and RO31-8220 resulted in a clearly reduced adhesion to endothelial cells (55% and 45% of control, respectively). Treatment of CCF-RC2 cells with GÖ6976 also led to a reduced cell adhesion (50%), whereas RO31-8220 treatment had no impact on cell adhesion in this cell line. The reducing effect of GÖ6976 on CCF-RC2 cells must be interpreted with caution, since our MTT test, which determines the influence of the inhibitor on cell vitality, also showed a slightly inhibitory effect of GÖ6976 in CCF-RC2 cells (Figure 1B). The distinct inhibitory effect of GÖ6976 in CCF-RC2 cells could partly be a result of the cell toxicity of this agent, causing a general reduction in cell vitality and consequently reduced cell adhesion. However, GÖ6976 reduced tumor cell adhesion to endothelial cells in both cell lines. The only PKC isoform which is inhibited by GÖ6976, but not by GF109203X, nor RO31-8220 or rottlerin, is PKCμ. PKD2 and PKCν (PKD3) are also inhibited by GÖ6076, although no isoform specific IC50 values for these PKD isoforms have been published. Because PKD2 and PKCν/PKD3 are not expressed in the cell lines tested, our results lead to the assumption that PKCμ is involved in the regulation of adhesion of the renal cancer cells CCF-RC1 and CCF-RC2.

PKCμ, also called PKD1 (protein kinase D), and additionally classified as a member of a new protein kinase family [23], has been shown to be activated dependent on PKCε or PKCη [37,38]. In cancer cells, PKCμ is known to be involved in metastasis. During this process, PKCμ forms a complex with paxillin and cortactin, leading to the formation of invadopodia, initiating cell migration and degradation of the basement membrane [39]. In this context, PKCμ mediated cortactin phosphorylation [40]. On the other hand, the loss of PKCμ expression increases the malignant potential of breast cancer cells [41]. Syed et al. showed that an overexpression of the subtype PKCμ1 in prostate carcinoma cells increased cell aggregation and decreased cell motility [42]. This PKC isoform has been shown to phosphorylate β-catenin, and consequently also influences cell motility [43]. In accordance with an important role in cell motility, PKCμ also mediates integrin recruitment to newly formed focal adhesions by promoting the recycling of αvβ3 integrin [44]. However, our study did not show any expression of β3 integrins in CCF-RC1 and CCF-RC2 renal cancer cells; therefore, this mechanism could not be studied.

In CCF-RC1 cells, treatment with GO6976 resulted in a reduced expression of β1 integrins, leading to the assumption that the effect of PKCμ on cell adhesion occurs via β1 integrins. In contrast, no reduced β1 integrin expression was detectable in CCF-RC2 cells. Assuming that this effect is not caused by a general reduced cell vitality induced by GO6976, and as detected by the MTT test performed in this study, a further cell adhesion mechanism seems to take place in this cell line. The adhesion of tumor cells to endothelial cells is also regulated by other cell adhesion molecules. The most significant integrin-independent mechanism between the interaction of tumor cells and endothelial cells is the binding of sialyl-Lewis × on the surface of tumor cells to E-selectin on endothelial cells. The expression of sialyl-Lewis × in tumor cells is regulated by members of the α1,3 fucosyltransferase (α1,3FuT) family, and in particular by FuT6 [45]. PKCμ has the ability to regulate the expression of FuT by inducing NFκB [46]. In this way, an inhibition of PKCμ should reduce the expression of sLeX on the RCC cells. Since the expression of sLeX on CCF-RC2 cells was less extensive compared to CCF-RC1 cells [47], these cells may be more sensitive to inhibition of sLeX. These mechanisms must be clarified in further investigations.

In CCF-RC1 cells, in addition to GÖ6976, RO31-8220 also reduced adhesion to endothelial cells. PKCε is reduced by RO31-8220 (IC50: 24 μM), but to a much lesser extend by GF109203X (IC50: 132 μM), and not by GÖ6976 or rottlerin. None of the PKC isoforms are inhibited by GÖ6976 and RO31-8220, and not inhibited by GF109203X or rottlerin at the same time (Table 1). Therefore, in CCF-RC1 cells, in addition to PKCμ, PKCε also seems to regulate the adhesion to endothelial cells. Iaska and coworkers described a functional relationship between PKCε activity and the membrane trafficking of β1 integrins in fibroblasts [48]. After activation, PKCε translocates to the cell membrane, where it colocalize with β1 integrins at membrane ruffles. Furthermore, inhibition of PKCε leads to accumulation of β1 integrins in vesicles, suggesting a PKCε dependent step in the trafficking of integrins [48]. Integrin trafficking is important for recycling after internalisation. Impaired PKCε dependent recycling reduces integrin expression on the cell membrane. For this reason, we analyzed the expression of integrins on the cell surface of CCF-RC1 and CCF-RC2 cells. And indeed, treatment with RO31-8220, in contrast to the other PKC inhibitors used, showed a strong inhibition of β1 integrin expression to 8% and 7% of control levels, respectively. This confirms earlier results showing a reduced β1 integrin expression after short time treatment of renal cancer cells with RO31-8220 [19]. Therefore, the deficient recycling of β1 integrins after treatment of tumor cells with RO31-8220 may offer an explanation for the observation made in the present study, i.e. that PKCε inhibition in both cell lines tested results in a reduced expression of β1 integrins on the cell membrane. Other authors also showed an involvement of PKCε in cell adhesion and proliferation. For instance, PKCε appears to be responsible for the regulation of HeLa cell adhesion to the extracellular matrix [20]. It is also implicated in β1 integrin-mediated adhesion of human breast carcinoma cells to collagen [49]. In mammary carcinoma cells PKCε as well as PKCμ have been shown to regulate cell adhesion to collagen IV in dependence of β1 integrins [44]. Furthermore, Engers and coworkers suggested a role of PKCε in invasion of renal carcinoma cells using a chick heart invasion assay [50]. However, in contrast to others, this study demonstrated a PKCε dependent adhesion to endothelial cells.

The two cell lines investigated differ in origin, although both were obtained from the same patient. CCF-RC1 was established from the primary tumor and CCF-RC2 from cells of the renal vein effluent of the perfused tumorous kidney [30]. Both have been characterized as renal carcinoma cells with similar characteristics, although CCF-RC2 cells have already performed the first step of metastasis, the detachment from the combined cell structure and migration from the primary tumor. For this reason, these cells are possibly more resistant against PKC inhibitors, which may explain their different behavior.

Although PKCμ and PKCε are known to influence cell proliferation [51-53], our investigations demonstrate a distinct alteration in cell proliferation solely after treatment of renal cancer cells with rottlerin, a PKCδ inhibitor. PKCδ is known to be involved in regulation of the cell cycle and apoptosis, and has also been shown to play a role in renal cell carcinoma [54]. PKCδ induces cell cycle arrest in many cell types by inhibiting the expression of cyclin D1 and cyclin E and up-regulating p27 [55-57]. Additionally, PKCδ activates DNA-PK (DNA dependent protein kinase) [58], antagonizes the Jak-STAT pathway and inhibits phospholipase D, an antagonist of Raf [59]. On the other hand, an inhibition of PKCδ induces apoptosis in B chronic lymphocytic leukemia cells, whereas in healthy B cells a more anti-apoptotic effect was mentioned [60]. In glioma cells an inhibition of PKCδ by rottlerin also led to a reduced activity of ERK and Akt, and inhibited cell proliferation [61]. These findings show a strong cell specific effect of PKCδ. Our results suggest a proliferation promoting effect of PKCδ in renal carcinoma cells.

## Conclusions

In conclusion, our results indicate that in CCF-RC1 and CCF-RC2 cells, PKCμ regulates tumor cell adhesion to umbilical vein endothelial cells. In CCF-RC1 cells, in addition to PKCμ, PKCε also regulates cell adhesion. Our observation of β1 integrin expression being regulated by PKCε, leads to the assumption that CCF-RC1 cell adhesion to endothelial cells is caused by affecting the expression of β1 integrins. In contrast, cell proliferation seems to be regulated by PKCδ.

## Abbreviations

BrdU: bromodeoxyuridine; CAMK: Calcium/calmodulin-dependent protein kinase; ECM: extracellular matrix; FAK: focal adhesion dinase; HUVEC: human umbilical vein endothelial cell; MTT: 3-[4,5-dimethylthiazol-2-yl]2,5-diphenyl tetrazolium bromide; PBS: phosphate buffered saline; DNA-PK: DNA dependent protein kinase; PKC: protein kinase C; PKD: protein kinase D; RCC: renal cell carcinoma.

## Competing interests

The authors declare that they have no competing interests.

## Authors' contributions

WB: study design, interpretation of the results, drafting of the manuscript. SB: cell adhesion experiments. ES: cell migration experiments. FB: flow cytometry analyses. REU: participation in flow cytometry analyses, interpretation of the results, critical revision of the manuscript. FR: participation in the study design, critical revision of the manuscript. JWT: participation in the study design, critical revision of the manuscript. CH: participation in the study design, critical revision of the manuscript

## Pre-publication history

The pre-publication history for this paper can be accessed here:

http://www.biomedcentral.com/1471-2407/10/183/prepub
